# Hypodermic Method of Injection

**Published:** 1869-06

**Authors:** 


Hypodermic Method of Injection.—The following is the *therapeutical portion* of the report of the committee on this subject of the Royal Medico-Chirurgical Society, a committee which embraced among its members such men as Savory, Henry Lee, Holmes, Durham, and its conclusions may be regarded as the best and most recent authoritative teaching we have in reference to hypodermic injections. We copy it from the thirty-second volume of the *Medico-Chirurgical Transactions*, London:

In this portion of their report the committee have drawn their conclusions as to the therapeutical advantages that distinguish the subcutaneous method of injection from experiments with a few active medicines, and though the list might have been extended, it must be borne in mind that many valuable drugs can not be used in this way, on account of the irritating properties they possess.

The intensity and the rapid sequence of effects which have already been shown to characterize the hypodermic method of administering drugs are important advantages, which are readily appreciated by the patient; and the dread which the slight operation may have caused at first is soon overcome when once the resulting benefits have been experienced.

In the relief of pain this method of introducing anodynes offers superior advantages to those in ordinary use; and in cases of delirium, of mania and of tetanus, where there is resistance or impediments to the ordinary modes of administering remedies, subcutaneous injection secures not only quickness of action, but also certainty as to the introduction of the drug.

Much difference of opinion exists on the question of localizing the injection. Cases have been communicated to the committee from which the superiority of local injection has been maintained; but although they have performed many experiments in reference to this question, the committee have failed to obtain any evidence to show that the local predominate over the general effects. They must therefore express their opinion that, though no symptom results from injection at the part affected, which is not shared equally by injections at any other part of the body, yet practically it may be advantageous to localize the injection for the sake of those effects upon the mind which localization will sometimes produce.

Injections may be repeatedly practiced in the same locality without any serious or permanent injury to the part. Mr. Roberts injected himself many times successively, in a very limited area, without any worse result than temporary thickening and irritation.

The committee have endeavored to procure details of untoward results following subcutaneous injection. One or two other cases have come to their knowledge, but of these they have been unable to obtain any satisfactory account.

The following are the results of their experiments on man in disease with aconitine, atropine and morphine:

*Aconitine.*—This drug was tried in three cases of neuralgia, but the local tingling which followed the injection was so severe that the drug was considered unfit for subcutaneous use. In one case, in which the neuralgia was of an hysterical character, the pain was relieved; in the other two cases, no alleviation was experienced. In the first case, 1-100th grain was used; in the others, 1-320th grain and 1-216th grain.

*Atropine.*—The anodyne properties of this drug are exhibited in a marked degree in subcutaneous injection.

In cases of simple neuralgia, atropine, when thus administered, is a very valuable remedy, and, in some cases, where morphine procured only temporary relief, the benefits derived from atropine injections were permanent. Very decided results were observed to follow minute doses of the drug used in this manner.

The pulse was accelerated to a considerable degree in one case, when 1-160th grain only had been injected. A larger dose should be given in cases of severe neuralgia, and the most satisfactory results were found to follow when decided toxical effects were manifested.

The discomfort (the excitement, the dry mouth, and the occasional disagreeable action on the bladder) experienced during the action of this drug presents a considerable hindrance to its general use. The cases in which atropine was used with advantage where cases of local neuralgia, lumbago and sciatica.

The initial doses are the eightieth of a grain for a woman, and the sixtieth for a man, but in cases of severe neuralgia larger doses may be given with safety. The largest dose mentioned to the committee was one-tenth of a grain.

*Morphine.*—The value of this drug is materially enhanced by this method of administration, and its action is not only secured with greater intensity and rapidity than by the ordinary modes, but the duration of its effects is prolonged, and some patients can tolerate it far better when it is injected under the skin than when it is given by the mouth.

Injected subcutaneously, this remedy does not invariably lose its virtue by repetition, and instances have come to the knowledge of the committee where the injection has been repeated daily for a number of years without the dose being augmented. Mr. Roberts expressly states that, though the injections were repeated in his own person more than a hundred times, the dose was never increased beyond two-thirds of a grain, and a smaller quantity was often found sufficient.

To confirmed opium-eaters, this method has been found of much service, smaller doses than those previously taken by the mouth being requisite. The largest dose mentioned by the committee was given to such a patient; as many as eight grains of the acetate were injected in this case.

Patients suffering from cancer have derived much benefit from the use of subcutaneous injections. Mr. Reeves mentions that from six to eight grains were injected in one case daily for a considerable period.

In allaying pain, the virtues of this drug are decidedly increased by injection, though the effects are not always permanent.

In cases of delirium tremens this method is often extremely useful, and in some instances were found to succeed where the introduction of the drug by the mouth failed; in a few instances, however, it seemed to have a negative result.

From the few cases of mania treated by injection that have come under notice, it would seem that this method of giving morphine is not altogether free from danger; in one case of mania the injection of half a grain proved fatal, and the same dose narcotized another patient for four days.

*The initial dose for an adult man, under ordinary circumstances, is from one-sixth to one-fourth of a grain; for a woman it should be smaller—from one-eighth to one-sixth.*

A few other cases where alarming symptoms have arisen from the injection of morphine have been forwarded to the committee. Briefly stated they are the following:

One-quarter of a grain in a man, not fatal.

Twenty-five minims of the liquor morphiæ acetatis, equivalent to five-twelfths of a grain of morphia, produced narcotism in a man, not fatal.

A quarter of a grain in a young lady twenty-four years of age, not fatal.

In those cases of mania already alluded to:

Half a grain in a woman suffering from acute mania, not fatal.

Half a grain in a similar case, fatal.

In some hospitals it has been the practice to inject a small dose of morphine after operations, for which chloroform has been used; the injection being made before the effects of the chloroform have passed off. It was stated that the sleep is prolonged by these means and the after effects of chloroform prevented, but from the experience of the committee on this point it would seem that the sickness following the use of chloroform is not always prevented by morphine injections, though it may be retarded.

